# A systematic review of training programmes for recruiters to randomised controlled trials

**DOI:** 10.1186/s13063-015-0908-6

**Published:** 2015-09-28

**Authors:** Daisy Townsend, Nicola Mills, Jelena Savović, Jenny L. Donovan

**Affiliations:** School of Social and Community Medicine, University of Bristol, Canynge Hall, 39 Whatley Road, Bristol, BS8 2PS UK

**Keywords:** Trials, Communication, Recruitment, Training, Randomisation, Equipoise

## Abstract

**Background:**

Recruitment to randomised controlled trials (RCTs) is often difficult. Clinician related factors have been implicated as important reasons for low rates of recruitment. Clinicians (doctors and other health professionals) can experience discomfort with some underlying principles of RCTs and experience difficulties in conveying them positively to potential trial participants. Recruiter training has been suggested to address identified problems but a synthesis of this research is lacking. The aim of our study was to systematically review the available evidence on training interventions for recruiters to randomised trials.

**Methods:**

Studies that evaluated training programmes for trial recruiters were included. Those that provided only general communication training not linked to RCT recruitment were excluded. Data extraction and quality assessment were completed by two reviewers independently, with a third author where necessary.

**Results:**

Seventeen studies of 9615 potentially eligible titles and abstracts were included in the review: three randomised controlled studies, two non-randomised controlled studies, nine uncontrolled pre-test/post-test studies, two qualitative studies, and a post-training questionnaire survey. Most studies were of moderate or weak quality. Training programmes were mostly set within cancer trials, and usually consisted of workshops with a mix of health professionals over one or two consecutive days covering generic and trial specific issues. Recruiter training programmes were well received and some increased recruiters’ self-confidence in communicating key RCT concepts to patients. There was, however, little evidence that this training increased actual recruitment rates or patient understanding, satisfaction, or levels of informed consent.

**Conclusions:**

There is a need to develop recruiter training programmes that can lead to improved recruitment and informed consent in randomised trials.

**Electronic supplementary material:**

The online version of this article (doi:10.1186/s13063-015-0908-6) contains supplementary material, which is available to authorized users.

## Background

The success of a randomised controlled trial (RCT) is in large part dependent on whether it manages to recruit the required number of participants so as to reliably answer the research question [1]. However, a review of 114 multicentre trials found that that only 38 % of RCTs achieved their original recruitment target [2]. Failure to meet recruitment targets can have important scientific, financial and ethical implications [3]. The research question may be compromised if the statistical power is weakened, the trial may need to be extended (thus increasing the workload and financial cost) or the trial may even be closed down prematurely, leading to a failure to address important healthcare topics.

Recruitment to RCTs is an interactional activity between a patient and trial recruiter (usually a doctor or nurse), which involves the provision of written information about the RCT and–unless the study is web-based–a discussion about whether to participate or not, in addition to information about diagnosis and treatment options [4]. Guidelines for Current Clinical Practice state that this information should include an explanation of the purpose of the trial, treatment options (including the standard treatment, uncertainty about experimental treatment arms, the features of each treatment arm, and potential risks and benefits), randomisation and the right to withdraw [5].

Patient information leaflets, which provide potential trial participants with written information on the trial and treatments, are strictly regulated by ethics committees but this does not extend to the verbal provision of information. Qualitative research has shown that information conveyed during recruitment appointments varies considerably in content and quality [6]. The communication style of the doctor or nurse introducing a patient to a clinical trial is suggested to be a key factor exerting an influence on patients’ preparedness to accept or decline participation into a trial [7]. For instance, one study found that discussions where physician communication built a sense of an alliance (among all parties, including family/companions), provided support (such as tangible assistance and reassurance about managing adverse effects) and provided medical information in understandable language, were associated with greater patient trial participation [8].

Although recruiters have a key role in patients’ decisions to participate in trials, a recent synthesis of qualitative studies has elucidated the range of practical and emotional challenges they can experience [4, 9]. Findings from interviews with recruiting staff from six RCTs showed that recruiters can struggle with explaining the rationale for RCTs to patients, being confident in admitting uncertainty, being willing to approach all eligible patients, eliciting patient preferences and exploring underlying reasons for them, and providing accurate information about the trial. These findings were supported by a systematic review of interventions to improve the recruitment activity of clinicians, which reported that many recruiters found it challenging to communicate about trials due to difficultly understanding and explaining concepts such as randomisation and equipoise [10].

A recent workshop on interventions to improve recruitment and retention identified training site staff as the number one priority for evaluation [11]. Although there have been systematic reviews on various interventions to enhance recruitment to RCTs (such as greater contact between trial coordinator and clinicians/trial sites, and comparing types of recruiters) [10, 12], none have focused exclusively on training for recruiters. Within these reviews only a handful of studies were identified that assessed the effectiveness of providing training for trial recruiters [13–16], with mixed results. Little is known also about how such training is being implemented (in terms of content, format and delivery), and how evidence of effectiveness is being evaluated. The aim of the current study was to systematically identify and review the available evidence across all study designs of the effectiveness of recruiter training interventions on recruitment to RCTs.

## Methods

### Criteria for inclusion of studies

#### Study types

All randomised, non-randomised or qualitative studies were eligible for inclusion. Due to the exploratory nature of the review, no studies were excluded by quality.

#### Participants

Health professionals and other trial staff involved in patient recruitment into RCTs.

#### Interventions

Training interventions delivered to trial personnel involved in patient recruitment into RCTs, with the aim of improving recruitment into trials or generally improving the success of trials, were eligible for inclusion. Any method (i.e. teaching packs, workshops) and mode (i.e. role play, presentation) of training was examined. Studies that evaluated only general communication training for health professionals not linked to RCT recruitment (i.e. the delivery of bad news) were excluded.

#### Comparison interventions

All types of comparison interventions (e.g. studies where training was compared to no training or a different training package) as well as studies without a comparison group were eligible.

#### Outcomes

The primary outcome of interest was host RCT recruitment rates. We also assessed the following outcomes when they were available: numbers of patients approached for recruitment to host RCTs, recruiter self-confidence, patient understanding of trial information and perceptions of recruiter communication, and observation of recruiter-patient trial consultations using pre-determined criteria.

### Search strategy

The review was conducted in accordance with Cochrane guidelines [17]. Studies were identified from Medline, Embase, CINAHL, the Cochrane Library and ERIC databases up until 14th July 2015. Search terms relating to recruitment, training and RCTs were combined to identify studies. Full search strategy is available (see Additional file 1). Reference lists of identified studies were also searched for further relevant studies.

### Selection of eligible studies

All titles and abstracts were screened for relevance by one researcher (DT) and full paper articles were obtained for records that were deemed relevant. Retrieved articles were read in full and assessed against the aforementioned eligibility criteria by DT. A second researcher (NM) then independently assessed all studies considered eligible or possibly eligible after first assessment and discussed with DT until agreement was achieved. Non-English papers were not translated due to lack of resources. Where more than one publication of the same study was found, the publication with the most complete data was included.

### Data collection and quality assessment

A data extraction form was developed specifically for the review to record details of each training programme (relating to authors, year, study design, participants, intervention (in terms of content, format and delivery), outcome measure(s) and results). Data were independently extracted by DT and NM. Judgements were compared and any areas of discrepancy were resolved by discussion amongst the two reviewers, and with the third author (JS) where necessary.

Quality assessments were performed using the Effective Public Health Practice Project (EPHPP) quality assessment tool for quantitative studies, which assessed studies by selection bias, design, confounders, blinding, data collection methods and withdrawals and drop outs [18]. The quality of qualitative studies was assessed using the Critical Appraisal Skills Programme (CASP) checklist, which covered rigour, key research methods used, credibility and relevance [19]. Quality assessment of all eligible studies were completed independently by two reviewers (DT and NM). Individual assessments were compared and any areas of discrepancy were resolved by discussion amongst the two reviewers and the other co-authors. Where necessary, corresponding authors were contacted to request further clarification regarding study details. Tables 1 and 2 show the agreed quality assessments.

**Table 1:** Quality assessment of the quantitative studies, using the EPHPP quality assessment tool

Study	Study design	Global quality rating	Study design	Protection against selection bias	Control for potential confounders	Blinding^a^	Reliability and validity of data collection methods	Retention
Bernhard et al. (2012) [27]	Randomised controlled	Strong	Strong	Strong	Strong	Moderate	Moderate	Strong
Kimmick et al. (2005) [14]	Randomised controlled	Moderate	Strong	Moderate	Strong	Moderate	Moderate	Weak
Hietanen et al. (2007) [28]	Randomised controlled	Strong	Moderate	Strong	Moderate	Moderate	Moderate	Strong
Kendall et al. (2012) [30]	Non-randomised controlled	Weak	Moderate	Weak	Weak	Weak	Moderate	Weak
Yap et al. (2009) [29]	Non-randomised controlled	Weak	Moderate	Weak	Moderate	Moderate	Moderate	Weak
Blazeby et al. (2014) [26]	Pre-test/post-test	Weak	Moderate	Weak	Weak	Moderate	Moderate	Weak
Brown et al. (2007) [23]	Pre-test/post-test	Weak	Moderate	Weak	Weak	Moderate	Moderate	Moderate
Donovan et al. (2009) [15]	Pre-test/post-test	Weak	Moderate	Weak	Weak	Moderate	Moderate	Weak
Fallowfield et al. (2012) [20]	Pre-test/post-test	Moderate	Moderate	Weak	Moderate	Moderate	Moderate	Moderate
Fallowfield et al. (2014] [25]	Pre-test/post-test	Weak	Moderate	Moderate	Weak	Weak	Moderate	Weak
Fisher et al. (2012) [21]	Pre-test/post-test	Weak	Moderate	Weak	Weak	Moderate	Moderate	Weak
Jenkins et al. (2005) [22]	Pre-test/post-test	Moderate	Moderate	Moderate	Weak	Strong	Moderate	Strong
Jenkins et al. (2013) [24]	Pre-test/post-test^b^	Moderate	Moderate	Strong	Moderate	Moderate	Moderate	Weak
Kenyon et al. (2005) [16]	Pre-test/post-test	Weak	Moderate	Weak	Weak	Weak	Moderate	Weak
Wuensch et al. (2011) [33]	Post training questionnaire survey	Weak	Weak	Weak	Weak	Weak	Weak	Weak

^a^ Blinding refers to outcome assessors only, as due to the nature of the intervention participants could not be blinded

^b^ The Jenkins study also included a randomised study comparing the influence of the duration of audit (12 vs 6 months before and after attendance of the training session) on recruitment success, which was not the focus of this review. Since all recruiters attended the training session (there was no comparison group without training) and outcome measures of interest for this review (patients approached and confidence discussing RCTs) were measured before and after the training was delivered, we categorised this study as uncontrolled pre-test/post-test design in the context of this review

**Table 2:** Quality assessment of the qualitative studies, using the Critical Appraisal Skills Programme (CASP) tool

	Study	
Criteria	Mann et al. (2014) [31]	Paramasivan et al. (2011) [32]
Global quality rating	Strong	Strong
Clear aims?	✓	✓
Qualitative methodology appropriate?	✓	✓
Research design appropriate to address aims?	✓	✓
Appropriate recruitment strategy?	✓	✓
Data collection appropriate?	✓	✓
Relationship between researcher and participants considered?	✗	✗
Ethic issues into consideration?	✓	✓
Data analysis sufficiently rigorous?	✓	✓
Clear statement of findings?	✓	✓
Valuable research?	✓	✓

### Data synthesis

We had planned to do meta-analysis of studies that have reported improvement in recruitment rates (our primary outcome) if sufficient number of studies with combinable outcomes were identified although it became evident that studies were too heterogeneous. Consequently, all outcome measures (including recruitment rates, numbers of patients approached for recruitment to host RCTs, recruiter self-confidence, patient understanding of trial information and perceptions of recruiter communication, and observation of recruiter-patient trial consultations) were analysed descriptively.

## Results

### Overview

**Fig. 1:**
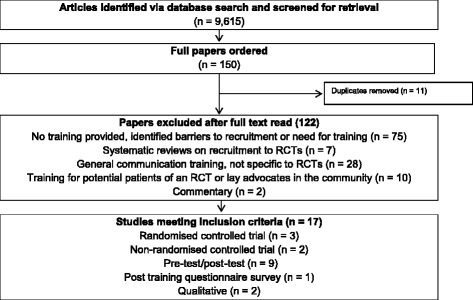
Study selection flow diagram

From 9615 titles and abstracts, 150 full articles were retrieved. Of these, 133 were excluded for not meeting the inclusion criteria or for being a duplicate publication, leaving 17 studies that were eligible for the review (Fig. 1). A summary of the included studies is available (see Additional file 2). Most studies evaluated their training intervention using an uncontrolled pre-test/post-test design (*n* = 9) [15, 16, 20–26]. Three were randomised controlled studies [14, 27, 28] and two were non-randomised controlled studies [29, 30], in which the experimental groups received a training intervention and control groups did not. Other designs included qualitative studies (*n* = 2) [31, 32] and a post training questionnaire survey (*n* = 1) [33].

Most studies (13/17) provided training in the context of cancer trials [14, 15, 20, 22–29, 32, 33]; the others were for diabetes [21], preterm labour [16], coronary heart disease [30], and orthopaedics [31]. Four studies had training interventions that focused only on recruitment to Phase III trials [22, 27, 30, 32], three had training sessions relating to phase I and II [20] or phase II and III trials [14, 23] and ten did not state the stage of the trial [15, 16, 21, 24–26, 28, 29, 31, 33]. In terms of the quality of the quantitative studies, most were deemed to be moderate (*n* = 4) [14, 20, 22, 24] or weak (*n* = 9) [15, 16, 21, 23, 25, 26, 29, 30, 33] with only two being classified as strong [27, 28]. Key weaknesses related to the potential for selection bias and confounders. Selection bias was most frequently related to the fact that participants were self-selecting, or were excluded from the intervention if they did not recruit sufficient numbers. Common confounders were whether participants had undergone any previous training (relating to RCTs or communication skills) and seasonal variations in host RCT recruitment rates, particularly in uncontrolled pre-post studies. The two qualitative studies were classified as strong in terms of the quality of the qualitative methodological approach, but they focussed more on the process of developing and implementing the training than an evaluation of its effectiveness [31, 32].

### Participants

Eleven of the studies stated participant study numbers [14, 20, 22–25, 27–29, 31, 33], and in these, 746 participants in total undertook recruitment training. However, six studies did not state the number of participants [15, 16, 21, 26, 30, 32]. Five studies provided information about participants’ previous communication training [22, 24, 27, 28, 33], and of these, 68 % were described as having had some form of previous general communication skills training. Two studies had recruiters who withdrew or were excluded from training as they did not enrol a sufficient number of patients [23, 27].

The majority of studies involved participants from a mix of disciplines, including research nurses [20, 22, 24–26, 28, 31], physicians [14, 22, 24, 25, 28, 29, 33], oncologists [20, 23–27], surgeons [24–26, 33], specialist nurses [24, 25], a trial data manager [19], midwives [16], gynaecologists [27, 33], radiologists [24, 25, 27], research assistants [20], histopathologists [24, 25], radiographers [22, 24], administrative staff [24], an MDT coordinator [25], and ‘other’ staff [24]. Five training sessions consisted of participants from a single discipline only [16, 20, 23, 29, 31]. Three studies did not provide information about participants [15, 30, 32].

### Training sessions

#### Training content

Most training programmes included both generic (e.g. key principles of RCTs) and specific (e.g. the evidence for a particular RCT) issues. The majority of training sessions focused on how to interact with patients, specifically with regards to the structure of RCT discussions with patients [15, 20, 22–29, 31, 32]. In addition, training programmes addressed avoiding coercive wording/particular terms [15, 23, 26, 27, 29, 32], discussing patient preferences [15, 22, 26, 31, 32] and strategies to optimise patient understanding [20, 23, 26, 29, 31]. Training also included ways to disclose controversial information [23, 27], explain the purpose of the trial [15, 20, 26], answer commonly asked questions [16], and enable patients to express concerns [32], as well as training on improving the quality of informed consent [28, 31, 32].

Several training sessions included information on recruitment pathways and how to approach potential trial participants [14, 16, 24–26, 32]. Key RCT concepts and information, such as uncertainty [15, 22, 32], eligibility criteria [32], randomisation [15, 24–26, 29], equipoise [15, 26, 32], the literature on recruiting strategies [25, 31] and trial-related burdens/barriers for patients [20, 21], multi-disciplinary team involvement [24, 25], trial management [25, 32] and how to encourage other staff to recruit [16, 25], were only included in some programmes. Some training also included general communication skills [21, 25, 29, 31, 33] or theories of communication [21, 23, 27, 33], including a shared decision-making framework [23, 27].

Training sessions that provided trial-specific information focused on the evidence and background to the study [16, 29, 31], how to give information on prognosis, care, and treatment risks and side effects for the condition under study [20]; and dealing with the psychological reaction to somatic disease [28], the effect of co-morbidity on cancer treatment [14] and toxicity in older patients [14]. Information was also given on problems specific to adjuvant trials [22], palliation trials [22], and those involving older cancer patients [14]. One study did not state the content of training [30].

#### Format and delivery

Training sessions mostly consisted of face to face workshops [14–16, 20–25, 27–33] held over one [14, 23, 25, 27, 29] or two [16, 20, 22, 24, 28, 33] consecutive days. Four programmes included training workshops over a longer period of time [15, 21, 31, 32]. Training workshops included group discussions [15, 20, 22, 24–26, 28, 31, 33], presentations/lectures [14, 15, 20–25, 27–29, 32, 33], videos [14, 20, 22, 23, 27, 33], listening to audio recordings [29, 31], role play [15, 21–25, 27–29, 31, 33], and personalised feedback [15, 23, 25–28, 31–33]. Six used simulated role play with actor ‘patients’ [20, 22–25, 33], and one study used the participating research nurses to act as patients in the role play [28].

In six studies the training was supplemented with further training in the form of post workshop individual feedback/discussions [15, 16, 21, 31–33], teleconferences [32], study days [16], or half day booster sessions [29]. Others provided additional aspects, including supporting written documents [14, 15, 20, 22, 23, 27–29, 32, 33], follow-up calls [16, 27], mugs and posters [16], and a thank you letter each time a patient was recruited [16]. In one study, training was supplemented with audit of recruitment practices in the period before and after the training, to evaluate if the knowledge of being audited affected recruitment success [24].

### Effectiveness of training

#### Evaluation methods

The effectiveness of training was determined by assessing recruitment rates to a host RCT [14–16, 21, 26, 30], the number of patients approached about trial participation [24], recruiters’ self-confidence in communicating with patients about trials [20, 22–25, 31], and patients’ understanding of the information provided in consultations and their satisfaction with recruiter communication skills [20, 22, 23, 27–29]. In addition, recruiter-patient consultations were observed for evidence of improvements in information provision and communication with patients, via audio [20, 23, 29, 31] and video recordings [22]. Recruiter feedback on the training intervention was also collected in a number of studies [20, 24, 25, 28, 31–33].

#### Recruitment rates and numbers of patients approached

Of the studies that assessed the impact of training on host trial recruitment rates and numbers of potential trial participants approached, there was no consensus as to whether training had an impact or not. Most uncontrolled studies found that there was a significant increase in recruitment rates after training had been provided compared with before [15, 16, 21], although one pre-test/post-test study found no significant difference in the rate of approaching patients post workshop [24]. Another uncontrolled study reported that before recruiter training, no patients were randomised. Following training, one centre randomised 5 of 16 eligible patients whilst the second centre identified no eligible patients before the end of the study [26]. A non-randomised study reported that patient recruitment rates to a host trial were significantly higher in sites involved in the training program than those that were not [30]. However, the only randomised controlled study to assess this outcome found no significant difference in patient accrual in the host RCT between those that had received the training compared with those that had not [14].

#### Recruiters’ self-confidence in communicating about trials

Four uncontrolled studies reported that recruiters’ self-confidence in communicating with patients about trials had significantly increased in all assessed areas after the training course [20, 22, 24, 25]–areas included explaining about randomisation [22, 24, 25] and the unlikelihood of personal medical benefit and a discussion of prognosis [20]. One of these studies reported that 5/6 team leaders felt that positive improvements had been maintained 6 months after the workshop, in that more team members were willing and able to discuss trials with patients [25]. However, another study reported no significant changes in recruiters’ satisfaction with the amount, clarity and completeness of information they provided, their ability to involve the patient in decision-making, and the perceived level of patient understanding [23]. One qualitative study continued to provide training until all recruiters stated that they felt more confident at communicating about trials with patients [31].

#### Patient understanding of information and satisfaction with recruiter communication skills

Evaluations of the effectiveness of recruiter training on patient understanding of information and satisfaction with recruiter communication skills produced mixed results. One randomised controlled study found no significant difference between patients’ level of satisfaction or decisional conflict between those who had consulted with the trained or control recruiters [27], although other controlled studies did report significant differences. For instance, one randomised controlled study found that patients reported a better understanding of the study aims if they had a consultation with recruiters who had received the intervention compared with those who had not [28], whilst a non-randomised controlled study found significant differences in patients’ understanding of information, such as greater understanding that participation in an RCT was voluntary [29]. However, these studies found no differences in other aspects of patient ratings, including understanding of randomisation [29] and the benefits of joining a trial [28]. Mixed results were also found between the pre-test/post-test studies, in that some aspects of patient outcomes in the post-training cohort had improved, including patients’ perceptions that recruiters were more likely to explain that trial entry was voluntary [20, 22] and having a more positive attitude towards clinical trials [23]. However, patient understanding of the trial remained unchanged [20] and they still had unanswered questions [22].

#### Assessment of recruiter-patient consultations

In several studies, recruiter-patient consultations were observed in practice [23, 29, 31] or in role play [20, 22] and assessed by the research team evaluating the effectiveness of training in relation to checklists of key concepts. A non-randomised controlled study reported that trained physicians communicated better with patients than untrained physicians in almost every assessed criteria, including that they tended to elicit parental questions and understanding in an open-ended way more frequently, and were better at clarifying parents’ questions or comments [29]. A further study employing qualitative methods found that some trial information (including assuring patients of confidentiality or that trial participation would not affect their current or future medical care) had been occasionally omitted during recruitment interviews prior to training, although following training these omissions no longer occurred [31].

Studies utilising a pre-test/post-test design reported that some aspects of recruiters’ communication skills when discussing clinical trials with patients had significantly improved following training, for instance, recruiters more commonly described the rationale or process of randomisation better [22, 23] and established patients’ understanding of various concepts [20, 22]. However other aspects, such as providing more information to patients [23] and informing them that they could withdraw at any time [20, 22], remained unchanged.

#### Recruiters’ feedback on the training interventions

Where assessed, the training interventions were perceived positively by the trial recruiters. In several studies recruiters provided feedback stating that they had found the training very useful [20, 24, 28, 33], beneficial [31, 32], interesting [20], would want to repeat the process in subsequent trials [31] and would recommend the workshops to other cancer trial teams [24, 25]. In particular, they reported positively on the usefulness of engaging in role play [24, 25, 28, 33], usefulness of group discussion [20, 33], receiving feedback [33], the informativeness of the workshop [20], the small size of the group [28], the secure and calm atmosphere [28], trial planning and facilitation [24, 25], quality of DVDs [20], relevance of topics [33] and the constructive learning environment [33]. However, it was suggested that training could have continued for longer or included another session of role play [28].

## Discussion

### Summary of findings

Although it is often stated that there is a need to provide training to those recruiting patients in trials [4, 6, 9–12, 34], only a small number of training programmes were identified in this systematic review. Most training programmes were uncontrolled observational studies of moderate or weak quality. Training was most commonly found in the context of cancer trials and tended to consist of workshops with a mix of health professionals over one or two consecutive days covering both generic and trial specific issues. The effectiveness of training was assessed by various measures including recruitment rates, numbers of patients approached for recruitment to host RCTs, recruiter self-confidence, patient understanding of trial information and perceptions of recruiter communication, and observation of recruiter-patient trial consultations using pre-determined criteria. Findings suggest that RCT recruiter training programmes are acceptable to recruiters and may increase their self-confidence and communication of key RCT concepts to patients. Studies with less robust study designs also suggested that training has the potential to improve recruitment rates and aspects of patient satisfaction and understanding of RCTs. However, the review found limited high quality evidence of interventions aimed at recruiters and therefore demonstrates the need to develop more robust designs to develop an evidence base on how best to target this group for training in trial recruitment. More comparative studies, especially randomised or clustered randomised trials, are the ideal method to assess the effectiveness of such training programmes.

Feedback from recruiters in several studies suggested that they had found the training intervention useful and evidence from a number of non-randomised and qualitative studies showed recruiter confidence in communicating trial information to patients had improved following training. Such findings are encouraging as previous research has highlighted that many find this challenging [9, 10]. Despite this, it appeared that recruiters still struggled with the amount, clarity and completeness of information to provide and had difficulty with explaining key RCT concepts such as randomisation. It is therefore perhaps not surprising that some studies reported no differences in patient satisfaction and understanding of trial information, or in host RCT recruitment rates, so more work is needed to address these identified training needs.

Due to the variation in content, delivery and format of training sessions it is difficult to determine precisely what is and is not effective at improving the process of recruitment to trials. Most of the training included presentations, videos, group discussions and role play. Feedback suggested that role play appeared to be particularly useful for recruiters as it was felt to provide a learning environment to apply new strategies and an opportunity to receive constructive feedback. Conversely, previous research shows that didactic based learning may not necessarily be most effective for learning [35]. In line with this, a randomised controlled study which used only didactic methods of delivery found no difference in trial recruitment rates between intervention and control groups [14].

The period of time required for training is likely to differ according to the experience of the recruiters and the complexity of the trial [31]. In the studies identified, the majority of training sessions ran over one or two days. Two studies, which utilised a randomised controlled and an uncontrolled pre-test/post-test design, found limited effects of a one day intervention and suggested that recruiters may have benefited from more training [23, 27]. There is some suggestion of a dose–response effect based on studies of general communication skills [35], although minimal training duration for a sustainable effect is unclear [27]. In the current review, one study which held multiple training sessions over a period of time found that recruitment rates increased from 65 to 81 % [15]. However, another study had training which lasted only 4 h in total and reported that recruitment increased from 43 to 58 % [21]. Bearing in mind the limitations of these observational uncontrolled designs, this suggests that content and delivery may be more important than length.

The majority of training workshops provided general information about the key principles of RCTs and also how to discuss trial concepts with patients. The interventions which appeared to have limited impact on outcomes did not address communicating with patients about concepts such as equipoise, randomisation, uncertainty, or exploring patient preferences [14, 23, 27]. As research has demonstrated that these are key issues that recruiters can find particularly challenging [4, 9, 10], it seems likely that training interventions which address these topics would be most beneficial to the experience of recruitment for recruiters and patients.

Research has suggested that recruitment training should address both generic and study specific skills [31]. According to deSalis and colleagues, ‘each RCT has a unique–and uniquely complex–recruitment pathway and its own set of issues that need to be resolved’ ([36] p.95). It therefore appears important to address issues specific to a particular trial, such as treatment arms, pathways or side effects, so that recruiters can discuss them confidently with patients, in addition to more generic trial recruitment issues. Fletcher et al. reported that the most successful interventions were studies which used qualitative research to identify the key training topics and then develop interventions based on this to improve recruitment [10].

Most of the studies in this review provided training for a mix of health professionals. Donovan and colleagues suggested that nurses and doctors who recruit to randomised trials experience different issues within their roles [4, 9]. It was recommended that doctors could benefit from support in relation to assessments of eligibility and equipoise [4], whereas nurses could benefit from support pertaining to perceived conflicts in their roles as recruiter, patient advocate and clinician, and helping them to be comfortable with approaching all eligible patients [9]. This suggests that training programmes may need to be targeted to the needs of different health professionals separately.

### Methodological considerations

This review was written in accordance with PRISMA guidelines to ensure that it was reported fully and transparently [37]. To minimise the risk of bias and errors, data extraction and the quality assessment of each study was performed independently by two researchers and disagreements were resolved by reaching a consensus through discussion with the third author if necessary. A wide range of sources were searched to identify training interventions on recruitment to RCTs for health professionals and trial staff recruiting to clinical trials, and a range of study designs were included. However, there is always the possibility that studies may have been missed as the search strategy was heavily reliant on text word searching, thus limiting searches to the terms used by authors in the title and abstract fields of each reference [38]. As non-English papers were not included due to lack of resources, there may have been studies in other languages that were missed.

The studies reviewed were of varying quality, limiting the conclusions that can be drawn. Two studies excluded participants from the training session as they did not recruit sufficient numbers [23, 27], although it could be argued that these individuals may have benefited most from training and support. Furthermore, six studies used simulated role play with actor ‘patients’ [20, 22–25, 33], and one study used participating research nurses to act as patients [28]. Whilst this may represent a more convenient option, it is possible that the simulated patients were not representative of ‘real’ patients. However, a study comparing role plays of trial recruitment discussions with real patients and simulated patients found that the latter were better informed about the purpose of a consultation and provided more specific feedback [39].

Relatively few studies investigated the effect of training on recruitment rates, making it difficult to draw conclusions as to how training could translate into practice. General communication training has been found to influence communication style in a clinical setting if both competence and self-confidence are improved [40]. Future research should use a range of measures to accurately understand effects of recruiter training, including the impact on recruitment rates, but ensuring also the capacity to assess levels of informed consent and to capture improvements in the ‘quality’ of the recruitment to trial consultation for both patients and recruiters. The interventions reviewed in this study focused mostly on training for the consultation in which trial recruitment is discussed. There are other issues outside of this consultation where training in trial recruitment might be beneficial, such as the assessment of eligibility, screening logs and recruitment pathways [9], which would benefit from further research.

## Conclusion

This review has identified a number of training programmes aimed at health professionals and trial staff who recruit to randomised controlled trials and assessed the evidence of the impact of training on the trial recruitment process. There is evidence suggestive of recruiter training programmes improving recruiters’ self-confidence and communication of some key RCT concepts to patients, but there is a wider gap in the evidence on the impact on recruitment rates and patient understanding of RCTs, satisfaction with recruiter communication and informed consent. Due to the limited quality of the evidence and the variation in interventions in terms of content, delivery and outcome measures, it is difficult to determine how training should best be implemented. Future research could develop recruiter training programmes based on topics identified from qualitative studies, and evaluate them using robust methods, so that it can be determined what kinds of training and support can improve recruitment rates while maintaining high levels of informed consent.

## Additional files

Additional file 1:
**Search strategy.** (DOCX 13 kb) Additional file 2:
**Summary of included studies.** (DOCX 24 kb)

## Abbreviations

CASP: Critical Appraisal Skills Programme
EPHPP: Effective Public Health Practice Project
RCTs: Randomised Clinical Trials (used only in reference to host clinical trials or RCTs in general terms, not to describe study designs used to assess the effect of training on recruitment success, which were always spelt in full)
