# Supplementary material for: A systematic review of training programmes for recruiters to randomised controlled trials
Source: Trials. 2015 Sep 28;16:432. doi: 10.1186/s13063-015-0908-6 (PMC4587840; doi:10.1186/s13063-015-0908-6)
Supplement: Additional file 1: — Search strategy. (DOCX 13 kb) [file 13063_2015_908_MOESM1_ESM.docx]

**Search strategy**

**Medline** (Date searched 14/07/15)

1. recruit*.mp.

2. train*.mp.

3. 1 or 2

4. RCT.mp.

5. trial*.mp.

6. 4 or 5

7. 3 and 6

**Embase** (Date searched 14/07/15)

1. recruit*.mp.

2. train*.mp.

3. 1 or 2

4. RCT.mp.

5. trial*.mp.

6. 4 or 5

7. 3 and 6

**CINAHL** (Date searched 14/07/15)

S1 recruit*

S2 train*

S3 S1 or S2

S4 RCT

S5 trial*

S6 S4 or S5

S7 S6 and S7

**Cochrane Library** (Date searched 14/07/15)

#1 recruit*:ti,ab,kw

#2 train*:ti,ab,kw

#3 #1 or #2

#4 RCT:ti,ab,kw

#5 trial*:ti,ab,kw

#6 #4 or #5

#7 #3 and #6

**ERIC** (Date searched 14/07/15)

S1 recruit*

S2 train*

S3 S1 or S2

S4 RCT

S5 trial*

S6 S4 or S5

S7 S6 and S7
