# Supplementary material for: A systematic review of training programmes for recruiters to randomised controlled trials
Source: Trials. 2015 Sep 28;16:432. doi: 10.1186/s13063-015-0908-6 (PMC4587840; doi:10.1186/s13063-015-0908-6)
Supplement: Additional file 2: — Summary of included studies. (DOCX 24 kb) [file 13063_2015_908_MOESM2_ESM.docx]

Table 1: Summary of studies included in the systematic review

| **Study** | **Design** | **Participants** | **Intervention** | | | **Measure of effectiveness and findings** |
| --- | --- | --- | --- | --- | --- | --- |
|  |  |  | **Included content** | **Format** | **Delivery** |  |
| **Bernhard et al (2012) [27]** | Randomised controlled | 62 oncologists | Ensuring a shared decision making framework  Structuring information into a sequence or order  Ensuring the inclusion of different and specific types of information in a clear manner  Consider the disclosure of specific controversial information and avoid coercive communication | 7 hour workshop | Role play  Video  Feedback  Presentation  Strategy document  Follow up phone call | Training had no significant effect on patient decisional conflict, satisfaction with consultation or satisfaction of doctors’ communication skills. |
| **Kimmick et al (2005) [14]** | Randomised controlled | 101 participants signed in, 29 signed at seminar | Older cancer patients  The effect of comorbidity on cancer treatment  Toxicity in older patients  Identifying patients for trials | Workshop  Monthly emails/letters | Presentation  Video  Supporting documents | No significant difference in patient accrual between control and intervention group (36% vs. 32% in first year, 31% versus 31% in second). |
| **Hietanen et al (2007) [28]** | Randomised controlled | Seven physicians and research nurses | The psychological reaction to somatic disease  Interviewing techniques  Patients’ needs when receiving information about a clinical trial  Quality of informed consent | Workshops over one evening and one morning | Presentation  Group discussion  Role play  Supporting written documents | Patients seen by recruiters in intervention were significantly more satisfied with consultation, better aware of the main objective of the study, recalled more therapeutic options outside of the trial were offered, and considered time given for making their decision sufficient more often than those of controls. However, patients in both groups still had misconceptions  Recruiters liked small size of group, secure and calm atmosphere and good presentation of role play. Four of six recruiters said they felt training could have continued for longer or included another session of role-play. |
| **Kendall et al (2012) [30]** | Non-randomised controlled | Recruitment staff from 373 sites | The program included a close working relationship with study sites by visiting them to provide training about recruitment issues and education about the trial |  |  | Patient recruitment rates were significantly higher at visited sites than un-visited sites (1.13 vs. 0.89 patients per site per month). |
| **Yap et al (2009)[29]** | Non-randomised controlled | 43 physicians | Patient understanding of randomisation  Choice of trial participation amongst parents of children with newly diagnosed leukaemia  Concept of RCT  Emphasis on holding two appointments  Gauging parental understanding both cognitively and emotionally before moving on  General communication skills | Workshops (one day long) and half-day  booster sessions | Presentation  Listening of audiotaped appointments  Role play | Trained physicians followed the sequenced approach more often than controls.  Physicians at the sites that received training tended to elicit parental questions and understanding in an open-ended way and clarify parents’ questions more frequently than physicians at the control sites  No significant difference in parental understanding of randomisation. |
| **Blazeby et al (2014)**  **[26]** | Pre-test/post-test | Surgeons, oncologists and nurses from two recruiting centres | Equipoise and balancing the presentation of treatment arms  Describing randomisation  Examples of ‘good’ and ‘not so good’ information provision  Exploring patient preferences for a particular treatment |  | Individual and group feedback | Before recruiter training, no patients were randomised. Following training, one centre randomised 5 of 16 eligible patients whilst the second centre identified no eligible patients before the end of the study. |
| **Brown et al (2007) [23]** | Pre-test/post-test | 10 oncologists | Facilitating shared decision making  Optimal sequence of information provision  Strategies to optimise patient understanding  Disclosing controversial information and avoiding coercive communication | 1 day workshop | Presentation  Video  Role play  Feedback  Strategy document | Ratings of consultations showed significant improvements in three shared decision making strategies, two clinical and ethical information items more frequently and reduced coercive behaviour after training. No change in other criteria. No change in doctors’ self-evaluation. Patients reported more positive attitudes to trial, although other outcomes unaffected. |
| **Donovan et al (2009) [15]** | Pre-test/post-test | Research nurses | Order and content of presentation of treatments  Ways to express equipoise and randomisation  Accurate and early explanation of the purpose of the trial  Empowering recruiters to elicit and address patients’ treatment preferences  The need for RCTs  Randomisation as a reasonable method | 8 workshops over 5 years (each up to 2 days) | Presentation  Group discussion  Role play  Feedback  Circulation of recruiter ‘tips’ documents | Immediate acceptance of allocation rose from 65% to 81% over 4 years with maintenance of randomisation rates over 65%. The intervention had the greatest effect when intensified training was combined with the circulation of second document. |
| **Fallowfield et al (2012) [20]** | Pre-test/post-test | 17 oncologists, 29 research nurses, one trial data manager | Structuring trial discussions  Giving prognostic information  Checking patient understanding  Discussing care  Describing aims of the trial  Risks and side effects  Trial related burdens for patients | 8 hour workshop over 2 days | Presentation  Group discussion  Video  Bibliography of reading materials | Ratings of consultations showed recruiters significantly improved on aspects of communicating about trials (including discussing the aims of the trial, discussing or checking patient understanding of prognostic issues and discussing standard treatment and symptomatic and palliative care options alongside the trial). No changes in other aspects, such as discussing right to withdraw.  Recruiters’ confidence increased significantly across all fifteen areas and they rated the workshop highly.  Patients’ perceptions of recruiter communication significantly increased in some aspects (for instance, there was greater understanding that participation was voluntary although no change in understanding of the aims of the trial). |
| **Fallowfield et al (2014) [25]** | Pre-test/post-test | 80 healthcare professionals from six MDTs | Barriers and drivers to trials  Describing randomisation  Patient pathways  Patient eligibility criteria  Team building and interpersonal communication  Strategies for timely trial set up | 1 day workshop | Role play  Feedback  Group discussion  Presentations  Supporting written documents | Recruiting participants showed a significant increase in levels of confidence for discussing trials and explaining randomisation.  Six month questionnaire data revealed 5/6 teams were more willing and able to discuss trial(s) with patients.  Positive feedback for trial planning, facilitation and role play, and 99% would recommend workshop to other breast cancer teams. |
| **Fisher et al (2012) [21]** | Pre-test/post-test | Research assistants | Communication programme to defuse subject resistance by contextualising the patient’s experience – emphasis on anticipation, acknowledgement, standardise, accept and plan (AASAP)  Barriers to trial involvement  Empathetic and non-judgemental communication skills | 2 x 2 hour workshops  Weekly meetings | Role play  Presentation | Following training, there was a significant increase in number of patients recruited (43% to 58%). |
| **Jenkins et al (2005) [22]** | Pre-test/post-test | 101 health professionals  (33 clinicians, 68 research nurses) | Difficulties discussing RCTs with patients  Problems specific to adjuvant RCTs  Handling uncertainty  Dealing with uniformed and suspicious patients  Palliative RCTs  Handling patient preferences | 8 hour workshop, over 2 days | Presentation  Group discussion  Video  Supporting written documents | Assessments of consultations showed that after training, recruiters improved on aspects of communicating about trials, including explaining randomisation, checking patients’ understanding, discussing treatment options and side effects  Health profs’ confidence increased significantly across all areas  Patient perceptions of recruiters’ communication significantly increased in most aspects, although three remained unchanged (including having unanswered questions). |
| **Jenkins et al (2013) [24]** | Pre-test/post-test^[[1]](#footnote-1)^ | 251 team members from 23 teams | MDT involvement in trials  Clarification of best pathways for recruitment (including omissions or ambiguities in the PIL, strategies for timely trial set up, and improvements in understanding each team member’s actual or putative communication roles)  Trial management problems | 1.5 day workshop | Presentation  Group discussion  Role play | There was no significant difference in the rate of approaching patients about trials post workshop, although it was estimated that there was an improvement 22% higher regression coefficient.  There was a significant improvement in four aspects of trial involvement  Recruiters’ confidence when discussing trials significantly improved.  Positive feedback for trial planning, facilitation and role play, and 99% would recommend workshop to other cancer teams. |
| **Kenyon et al (2005) [16]** | Pre-test/post-test | Midwives from 79 sites | Evidence and background to the study  How to approach patients  How to encourage others to recruit  How to answer commonly asked questions | 2 day workshop  Study days at six monthly intervals, monthly calls and three monthly site visits |  | Recruitment significantly increased by an average of 69% in the 79 centres (range -89% to +200%) following introduction of training. |
| **Wuensch et al (2011) [33]** | Post training questionnaire survey | 40 physicians | Theoretical background of physician-patient communication  SPIKE protocol (Setting, Patient’s Perception, Information Need, responding to Emotions with Empathy and Summary) | Workshops (17 hours in total) | Presentation  Video  Group discussion  Role play  Feedback  Summary card | The workshop received positive feedback.  Practicing with actor patients and receiving constructive feedback was seen as most useful. |
| **Mann et al (2014) [31]** | Qualitative | 4 research nurses | Nurses familiarised themselves with trial information and literature on recruiting strategies  Nurses reviewed the interviews using a standardised checklist covering trial details and key concepts  Effective strategies of conveying key concepts, demonstrating informed consent, and ensuring voluntary participation were explored and adapted | Five workshops over 12 weeks, between 40-60 minutes | Literature reading  Role play  Group discussion  Individualised feedback | Initially felt anxious at prospect of interviews being judged by others.  Some trial information was occasionally missing from recruitment discussions, which no longer occurred after third review session.  All nurses felt that communication and recruitment abilities were enhanced overall and that they would want to repeat this process in subsequent trials  Meetings concluded when all the nurses felt competent at recruiting. |
| **Paramasivan et al (2011) [32]** | Qualitative | Recruitment staff | Summary of qualitative recruitment findings  Examples of ‘good’ practice  Patient eligibility  Equipoise  Address patient preferences  Strategies to enable patients to express concerns | Four workshops (two via teleconference)  Recruitment tips document | Individualised feedback  Presentation | Study was closed due to eligibility criteria, but qualitative data suggested that training sessions had been useful.  Recruiters felt more confident in managing patient treatment preferences. |

1. The Jenkins study also included a randomised study comparing the influence of the duration of audit (12 vs 6 months before and after attendance of the training session) on recruitment success, which was not the focus of this review. Since all recruiters attended the training session (there was no comparison group without training) and outcome measures of interest for this review (patients approached and confidence discussing RCTs) were measured before and after the training was delivered, we categorised this study as uncontrolled pre-test/post-test design in the context of this review. [↑](#footnote-ref-1)
